# Multimodal fusion and human-robot interaction control of an intelligent robot

**DOI:** 10.3389/fbioe.2023.1310247

**Published:** 2024-01-04

**Authors:** Tao Gong, Dan Chen, Guangping Wang, Weicai Zhang, Junqi Zhang, Zhongchuan Ouyang, Fan Zhang, Ruifeng Sun, Jiancheng Charles Ji, Wei Chen

**Affiliations:** ^1^ Institute of Intelligent Manufacturing, Shenzhen Polytechnic University, Shenzhen, China; ^2^ AVIC Changhe Aircraft Industry (Group) Corporation Ltd., Jingdezhen, China

**Keywords:** kinematic modeling, robotic walker, multimodal fusion, human-robot interaction control, stroke

## Abstract

**Introduction:** Small-scaled robotic walkers play an increasingly important role in Activity of Daily Living (ADL) assistance in the face of ever-increasing rehab requirements and existing equipment drawbacks. This paper proposes a Rehabilitation Robotic Walker (RRW) for walking assistance and body weight support (BWS) during gait rehabilitation.

**Methods:** The walker provides the patients with weight offloading and guiding force to mimic a series of the physiotherapist’s (PT’s) movements, and creates a natural, comfortable, and safe environment. This system consists of an omnidirectional mobile platform, a BWS mechanism, and a pelvic brace to smooth the motions of the pelvis. To recognize the human intentions, four force sensors, two joysticks, and one depth-sensing camera were used to monitor the human-machine information, and a multimodal fusion algorithm for intention recognition was proposed to improve the accuracy. Then the system obtained the heading angle E, the pelvic pose F, and the motion vector H via the camera, the force sensors, and the joysticks respectively, classified the intentions with feature extraction and information fusion, and finally outputted the motor speed control through the robot’s kinematics.

**Results:** To validate the validity of the algorithm above, a preliminary test with three volunteers was conducted to study the motion control. The results showed that the average error of the integral square error (ISE) was 2.90 and the minimum error was 1.96.

**Discussion:** The results demonstrated the efficiency of the proposed method, and that the system is capable of providing walking assistance.

## 1 Introduction

In 2019, there were an estimated 12.2 million cases of apoplexy (95% uncertainty interval (UI) 11–13.6 million) in the world, with an estimated 101 million sufferers according to the Global Burden of Disease Study (GBD et al., 2021). The increase in stroke patients has resulted in 143 million cases of disability-adjusted life-years (DALYs), and there are currently around 1.3 billion people with disabilities according to data from the World Health Organization (Feigin et al., 2022). Under this tough situation, the present healthcare system, lack of bridle-wise physiotherapists (PT), assistive technology, and effective rehabilitation equipment cannot meet the increasing demand for rehab training, and disability of the lower extremities limits functional independence in activities of daily living and significantly deteriorates the quality of life of the affected individual (Chen et al., 2020; Jarva et al., 2021; Lesaine et al., 2022). Studies have shown that robot-assisted rehabilitation training is more effective than traditional gait training in improving walking ability and balance functions in stroke patients (Nam et al., 2017; Capecci et al., 2019; Calabrò et al., 2021). Furthermore, rehab training using gait assistance could help in providing intensive therapeutic exercises while also allowing for a quantitative assessment of the recovery (Mirelman et al., 2019). However, the trial-and-error learning hypothesis in motor control research suggests that position-control-based movement might decrease motor learning for some tasks, and the human-robot interaction control is the main pain point of training aiding (Reisman et al., 2010; Mojadidi et al., 2017).

During normal walking with the robotic walker, the control system usually recognizes the human intentions via the interactive sensors, then outputs the actuating speed of the wheels based on the classification and interactions. In the literature focused on human-robot interaction (HRI) strategy for human mobility assistance, the cognitive Human-Robot Interaction (*c*HRI) and the physical Human-Robot Interaction (*p*HRI) with humans applied in wearable robotics are explained by Pons et al. (2008). The *c*HRI is explicitly developed to obtain the data acquired by a set of sensors to measure bioelectrical and biomechanical variables. Takanori O. et al. developed an assist robotic walker (JARoW-II) for elderly people, and proposed a pelvic-based walking-support control technique without the use of specific manual controls or additional equipment, via two laser range finders (LRFs) to obtain coordinate data for the surface of the user’s lower limbs (Ohnuma et al., 2017). The pHRI is based on a set of actuators and a rigid structure that is used to transmit forces to the human musculoskeletal system. For example, Sierra M. et al. developed Smart Walkers to improve physical stability and sensory support for people with lower limb weakness via a haptic joystick with three operational modes (Sierra M. et al., 2019), after that they proposed the AGoRA Smart Walker with a human detection system and a user interaction system, and the walker can estimate the intentions via thehuman–robot–environment interface (Sierra et al., 2019). However, the integration of classic Human-Computer interfaces (HCi) with newer types of interfaces facilitates effective interaction (Sharma et al., 1998), such as speech or visual interfaces, tactile sensors, the LRF, the IMU, and force/torque sensors. The ASBGO system proposed by the University of Minho is a typical example, the walker was equipped with load cells, an infrared sensor, the Inertial Measurement Unit (IMU), and a real sense camera to detect the postural and gait parameters of the user (Moreira et al., 2019). To improve the accuracy of the task, a new multimodal interface for walker-assisted gait is proposed, which involves the integration of different modalities (Frizera et al., 2011). The UFES’s smart walker combined force sensing and lower limbs monitoring to detect the user’s legs and showed accurate performance in all experiments (Valadão et al., 2016). However, multi-modality information fusion facilitates better use of the relationships between multiple types of data, which can improve the model matching accuracy and effectiveness (Cifuentes and Anselmo, 2016; Horii and Nagai, 2021; Su et al., 2023). Therefore, this paper proposed a method for the multimodal fusion and the HRI control, the video image from the real sense camera, the interaction forces from the load sensors, and the motion vector from the joysticks were employed to detect the interaction information, based on the multimodal fusion method, a new interactive controller was designed to assist the patients.

The remainder of this brief is organized as follows. Section 2 contains a description of the RRW system. Section 3 describes the modeling of the system, and formulation of the control problem as well as the design and implementation of the desired controller. Section 4 presents the setup and results of the preliminary test with three volunteers. Finally, Section 5 concludes the brief.

## 2 System description

**FIGURE 1:**
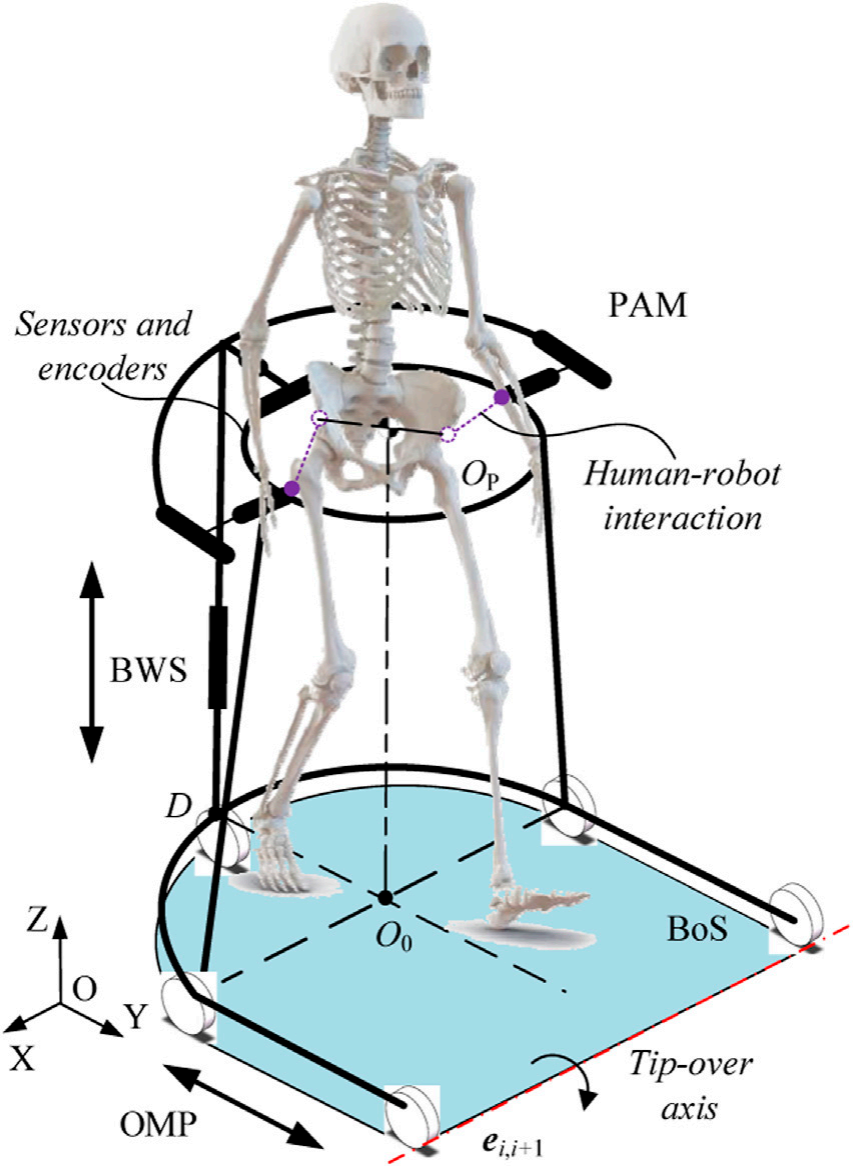
Conceptual model of system.

Generally, a human-robot interaction system works in conjunction with a mobile platform to achieve gait assistance, the robotic walker provides the user with a safe environment via balance maintenance, meanwhile, sensors and encoders are employed to detect the motion intention of the user and calculate the control output (Zhao et al., 2020; Wang et al., 2023). Therefore, as shown in Figure 1, we designed a robotic walker consisting of three main parts: i) an omnidirectional mobile platform (OMP); ii) a body weight support system (BWS), and iii) a pelvic assist mechanism (PAM), the design details will be described.

### 2.1 Hardware description

**FIGURE 2:**
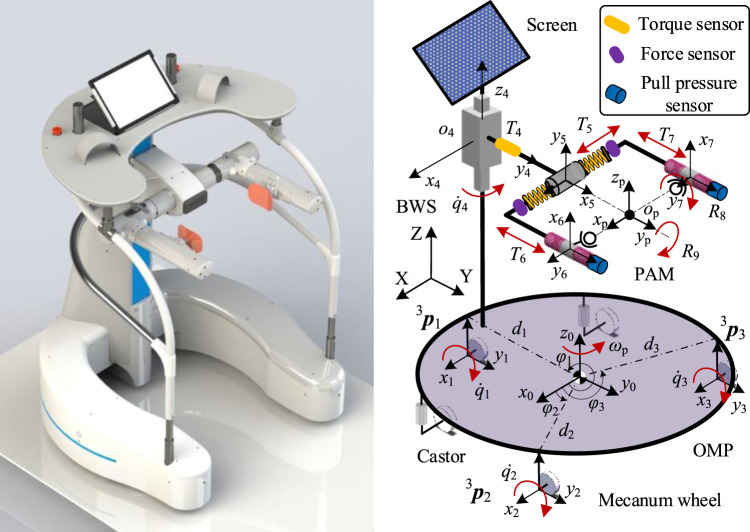
The CAD model of the walker and coordinate system.

In this paper, we present a walking assist system facilitating pelvic movements for several reasons. First, based on the walking characteristics of the patients and the problem definition, pelvic movement abnormalities lead to an increase in the double support phase and abnormal gait. Second, pelvic obliquity and pelvic rotation are the key parameters for lower extremity motor function. And third, the pelvic motions are associated with the gait. Therefore, we proposed the PAM to smooth the pelvic motions and install the force/torque sensors, as shown in Figure 2, so we can detect the middle-lateral and vertical displacement of the pelvis, as well as pelvic obliquity and pelvic rotation. Based on the range of pelvic motions during normal gait, the user can achieve normal gait with the help of the PAM. The *T*_5_ corresponds to the middle-lateral displacement, which consists of a set of ball splines and two springs, and the displacement is monitored by the force sensors at the end of the spring. Similarly, *T*_6_ and *T*_7_ are coupled and correspond to the forward-back displacement and pelvic rotation, as the pelvis is connected to the walker by the sliders of the two ball splines. The pose information of the pelvis is given through calculating sensor data. Then there is a revolute pair to achieve pelvic obliquity and tilt, labeled as R_8_ and R_9_ respectively. Furthermore, one torque sensor is installed on the joint pontes between the BWS and the PAM to detect the vertical motion.

For weight offloading and reduction of the cardiopulmonary burden, a servo motor is designed to realize the approximately 0.5 m vertical displacement of the pelvis and provide subjects with appropriate body weight support via a guide screw and a set of linear guideways. The error between the offloading value and sensor signal is used to trace the pelvic motion, and the system control is implemented in TwinCAT2 using a controller (Beckhoff PLC CX5130). On the top of the BWS, the control platform is installed to support the upper body weight and implement the interaction control. Two joysticks and one depth-sensing camera (Surface Go 2) were used to monitor the human-machine information, the user manipulated the walker via the left or right joystick according to the actual condition. The depth-sensing camera is used to obtain the facial features, as the heading angle can reveal the motion intention.

The primary aim of the OMP is to provide over-ground mobility, and thus achieve gait assistance. The OMP consists of three active wheels to provide power, two passive castors to maintain balance, and a U-shaped rigid steel frame to provide an installation base. According to the motions of lower limbs, a U-shaped rigid steel frame is designed to satisfy approximately 0.5 m of free space in the medio/lateral direction, and 0.8 m of free space in the anterior/posterior direction. For the active omnidirectional wheels, the walker is capable of rotation with arbitrary radius.

As described above, the multimodal Human-Robot Interaction (*m*HRI) is used to estimate the motion intention: the video image from the real sense camera, which belongs to the *c*HRI. the interaction forces from the load sensors and the motion vector from the joysticks were employed to detect the interaction information, which belongs to the *p*HRI.

### 2.2 Problem statement

In the interactive control process of lower limb rehabilitation robot, it is easy to produce more interference signals for the abnormal walking characteristics of the hemiplegic patients, which leads to the indisposed control performance, and then the robotic walkers cannot assist the users to finish the Activity of Daily Living (ADL) tasks. For the problem at hand, mobile rehab robots need to perceive the motion intentions via the *c*HRI or the *p*HRI. For passive walkers, the problem is to detect the safety of the user and brake at the right moment. For the active walkers, the HRI is more important for that the system needs to identify the gait pattern accurately and output appropriate velocity to trace the user. According to the above analysis, the two major issues are: 1) estimating the motion intentions; and 2) calculating output velocity.

For estimating the motion intentions, the JARoW-II active robotic walker obtained the coordinate data for the surface of the user’s lower limbs via the two LRFs (Hokuyo Automatic Co. Ltd. model URG-04LX), the advantage of the scheme is capable of the gait information acquisition, but the drawback is large amounts of computation; the KineAssist rehab robot estimated the interaction forces via two ATI force/torque sensors at both side of the pelvis, the advantage of the scheme is capable of the pelvic information acquisition, but the drawback is exorbitant price (Hurt et al., 2015). To sum up, there exist some problems with the current solution, such as high cost, poor intelligence, and inaccurate intention recognition.

For calculating output velocity, the mobile robots following behind a user is a common approach in walker-assisted locomotion (Seo and Lee, 2009), and it is more natural and comfortable for the person to control the walker if the robot is placed in front of the user (Haoyong et al., 2003). But the control problem is the position error between the mobile robots and humans, for the signal delay from the *c*HRI or the *p*HRI. The potential solution is to control the system to minimize the tracking error between humans and the mobile robot locomotion. A virtual spring model is used to absorb the gap between the human and the mobile robot motion via the input velocity generated on the basis of an elastic force (Morioka et al., 2004). But for *p*HRI, this solution can lead to a radical change in the interaction force.

**FIGURE 3:**
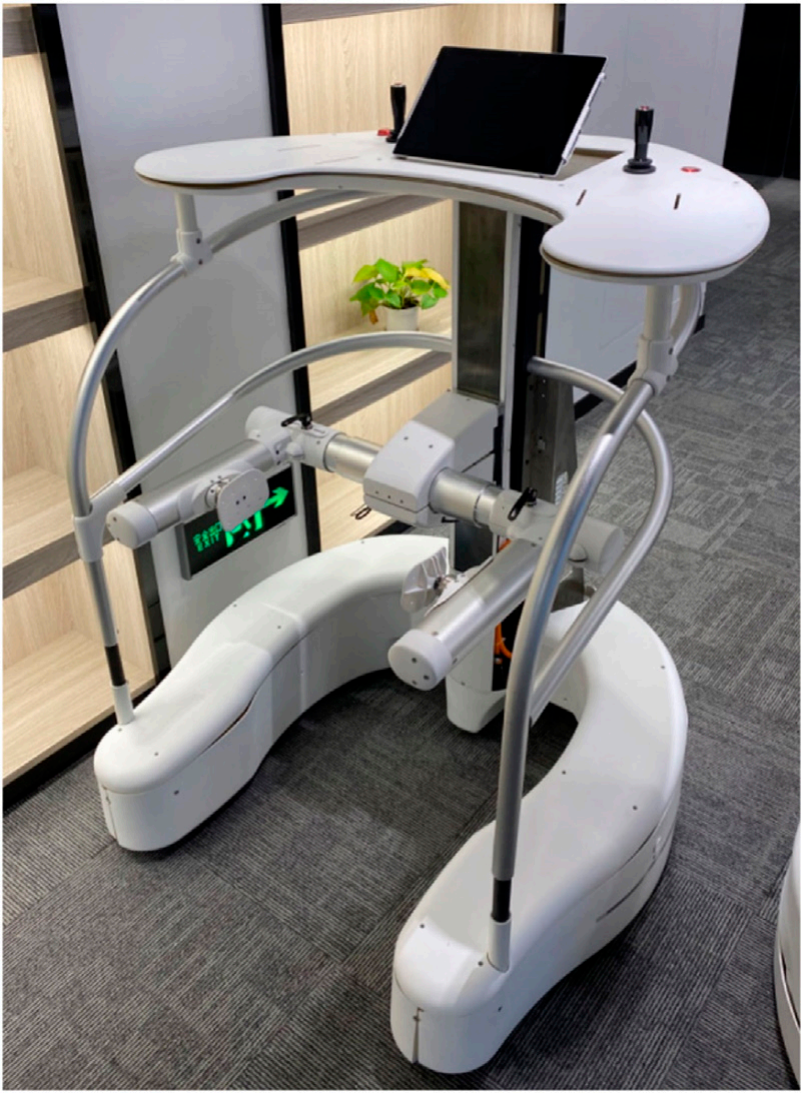
The physical prototype of the robotic walker.

Therefore, we present a method to obtain the heading angle *E*, the pelvic pose *F,* and the motion vector *H* via the camera, the force sensors, and the joysticks respectively, as shown in Figure 3, and go through several steps to get the classification of gait pattern, then output the velocity to minimize the tracking error, the methods are detailed in the next chapter.

## 3 Modeling and control

In order to improve the movement performance and the controllability of the robotic walker, the robot movement control model was produced in this chapter based on the robot kinematics, the active and passive joints were involved. The relation between the tacking velocity and the output of the servo motors was derived. On this basis, the control method was presented, which is two stages of control: the first step performed the *m*HRI detection via the camera, the force sensors, and the joysticks; while the second step corresponded to an inverse kinematic controller.

### 3.1 Kinematic model

The human-robot interaction model is shown in Figure 2, The variables and parameters used in this paper are defined as follows: the OXYZ is a global coordinate system, point $o_{0}$ is the center of the circle of the OMP, $o_{0}x_{0}y_{0}z_{0}$ is a local coordinate system attached to the robotic walker, three omnidirectional wheels are uniformly distributed along the circumference frame, with the center $o_{i}\left(i=1,2,3\right)$ , and $\varphi_{i}\left(i=1,2,3\right)$ represents the position angle of the three wheels. The BWS system is located at point *D*, through the pelvic assistance mechanism connecting to the pelvic center *o*_*p*_, point *C* is the mass center of the robotic walker. Using the position, orientation, and velocity of point $o_{0}$ to indicate the position, orientation, and velocity of the robotic walker, θ is the heading angle of the mobile platform relative to the *X*-axis. r is the radius of the driving wheel, s is the screw lead of the BWS’s precision ball screw, $\varphi_{i}\left(i=1,2,3\right)$ is the distance from the mass center *C* to the three omnidirectional wheels.

Defining the velocity matrix of the OMP relative to the global coordinate system as $\dot{q}={\left[\begin{array}{ccc} \dot{x} & \dot{y} & \dot{\theta } \end{array}\right]}^{T}$ , the angular velocity of the three driving wheels as $\omega ={\left[\begin{array}{cc} {\dot{\theta }}_{1} & \begin{array}{cc} {\dot{\theta }}_{2} & {\dot{\theta }}_{3} \end{array} \end{array}\right]}^{T}$ , and the velocity matrix relative to the local coordinate system as ${\dot{q}}_{R}={\left[\begin{array}{ccc} {\dot{x}}_{R} & {\dot{y}}_{R} & {\dot{\theta }}_{R} \end{array}\right]}^{T}$ . Under the circumstances that the kinestate of driving wheels is pure rolling without slide, and the mobile platform is able to do instantaneous motion along the heading direction of the driving wheels. Defining the velocity of the PAM, i.e., T_4_ relative to the global coordinate system as $\dot{z}$ . The angular velocity of the screw as ${\dot{\theta }}_{4}$ .

According to robot kinematics and the earlier paper (Ji et al., 2021), deducing the mapping relation $H\left(q\right)$ between the tracking velocity and angular velocity of the joints:

$$H\left(q\right):R^{n}\to R^{l}$$ (1)

where $R^{n}$ represents the set of the joint angle q , velocity $\dot{q}$ and accelerated velocity $\ddot{q}$ , and $R^{l}$ represents the set of the generalized position and posture vector x in the local coordinate system as defined in Eqs 2, 3:

$$x\in R^{l}$$ (2)

$$q,\dot{q},\ddot{q}\in R^{n}$$ (3)

Then, the velocity vector $\dot{x}$ in the local coordinate system can be obtained by the partial derivative about the mapping relation $H\left(q\right)$ , as:

$$\dot{x}=\left(\partial H\left(q\right)/\partial q\right)\dot{q}=J\left(q\right)\dot{q}$$ (4)

where $J\left(q\right)$ of Eq. 5 is the Jacobian matrix, which belongs to the set $R^{l\times n}$ ,

$$J\left(q\right)\in R^{l\times n}$$ (5)

According to the derivation of the earlier paper, for the active joints, the inverse Jacobian matrix can be obtained as Eq. 6,

$$J^{-1}=\frac{1}{r}\left[\begin{array}{cccc} -1 & 0 & 0 & d_{1}\cos\left(\pi /2-\varphi_{1}\right)\\ 0.5 & -0.866 & 0 & d_{2}\cos\left(\pi /6-\varphi_{2}\right)\\ 0.5 & 0.866 & 0 & d_{3}\cos\left(\pi /6+\varphi_{3}\right)\\ 0 & 0 & 2\pi r/s & 0 \end{array}\right]$$ (6)

Through the matrix inverse in the MATLAB, the explicit expression of the Jacobian matrix can be written as Eq. 7:

$$J\left(q\right)=r\left[\begin{array}{cccc} -2/3 & 1/3 & 1/3 & 0\\ 0 & -\sqrt{3}/3 & \sqrt{3}/3 & 0\\ 0 & 0 & 0 & 3s/2\pi r\\ \frac{1}{3d_{1}\cos\left(\pi /2-\varphi_{1}\right)} & \frac{1}{3d_{2}\cos\left(\pi /6-\varphi_{2}\right)} & \frac{1}{3d_{3}\cos\left(\pi /6+\varphi_{3}\right)} & 0 \end{array}\right]$$ (7)

**FIGURE 4:**
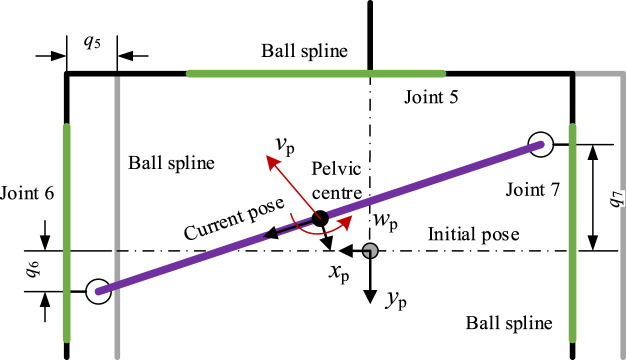
Mechanical construction of the PAM.

For the passive part of the robotic walker, as shown in Figure 4, the pelvic motions can lead to a change of position, so the D-H method is used to calculate the relation between the current pelvic pose and the passive joints, then we can obtain the current pelvic pose through the inverse kinematics and sensor data.

For the passive joint i, the rotation matrix Ri‐1i and displacement vector $b_{i}^{\prime }$ contain the static joint structure ( Ri‐1si and $b_{si}^{\prime }$ ) and dynamic rigid motion ( $R_{mi}$ and $b_{mi}^{\prime }$ ), which can be expressed as Eqs 8, 9:

Ri‐1i=Ri‐1siRmi (8)

$$b_{i}^{\prime }=b_{si}^{\prime }+b_{mi}^{\prime }$$ (9)

According to Eq. 1, the position vector and rotation matrix of the joint can be obtained as Eqs 10, 11:

P0n+1=∑i=0nR0ibi′ (10)

R0n=∏i=1nRi‐1i (11)

Via Eq. 4, a velocity vector in the local coordinate system can be obtained. To translate into the velocity vector in the global coordinate system, taking point $o_{0}$ as a reference point, Eq. 4 premultiplies rotation matrix and $t_{R}={\left[\begin{array}{cc} v_{R} & \omega_{R} \end{array}\right]}^{T}$ translates to robot velocity in the global coordinate system $t_{M}$ :

$$t_{M}=J_{G}t_{R}$$ (12)

where $t_{M}={\left[\begin{array}{cc} v_{M} & \omega_{M} \end{array}\right]}^{T}$ , $J_{G}$ is the rotation matrix. Then we can obtain the velocity and angular velocity of the robotic walker in the global coordinate system, which is used for error tracking.

### 3.2 Intention prediction

**FIGURE 5:**
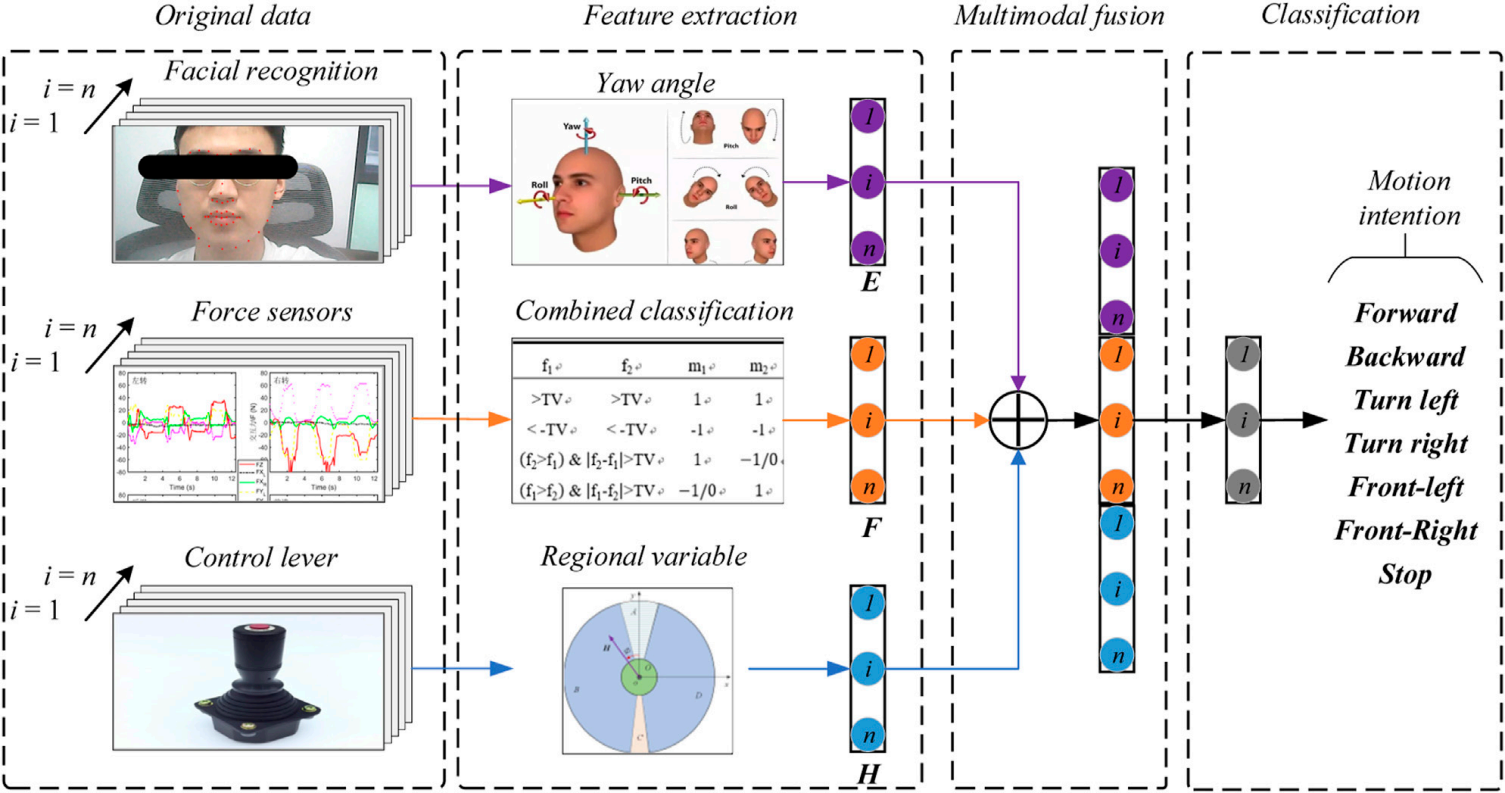
The multimodal human-robot interaction model of the robotic walker.

The multimodal human-robot interaction model is shown in Figure 5, the facial recognition is the *c*HRI, which is used to detect the Yaw, Roll, and Pitch angle of the head via a real sense camera, then we can predict the direction the user wants to go and extract the angle feature Ε . The force sensors and control levers are the *p*HRI, which are used to interact with users. The force sensors installed on the PAM can not only able to obtain the interaction force and torque but also detect the current pelvic pose. By the signal combination of the sensors and the principles of human motion, the motion intentions of the lower limbs can be obtained as F . Secondly, the control levers are used to interact with the hands, and we can obtain the motion intentions by the information vector, defined as Η . After the feature extraction, we combine the feature via multimodal fusion, which classifies the motion intention into seven types: forward, backward, turn left, turn right, front-left, front-right, and stop.

For the control levers, we can control the walker with two levers as described in the previous paper (Ji et al., 2021). This paper addressed the prediction pattern with a single control lever, defining the lever vector as H , the magnitude of the vector as $\left|H\right|$ , the direction of the vector as Φ , and the method to obtain the motion intentions is as follows:

1) Classify the workspace of the lever into five regions OABCD, defined as Eqs 12a–16:

$$O\in \left|H\right|\leq t,-\pi <\Phi \leq \pi$$ (12a)

$$A\in t<\left|H\right|\leq m,-\pi /12\leq \Phi \leq \pi /12$$ (13)

$$B\in t<\left|H\right|\leq m,-17\pi /18<\Phi <-\pi /12$$ (14)

$$C\in t<\left|H\right|\leq m,17\pi /18\leq \Phi \leq -17\pi /18$$ (15)

$$D\in t<\left|H\right|\leq m,\pi /12<\Phi <17\pi /18$$ (16)

where t is the threshold value, m is the maximum value.

**FIGURE 6:**
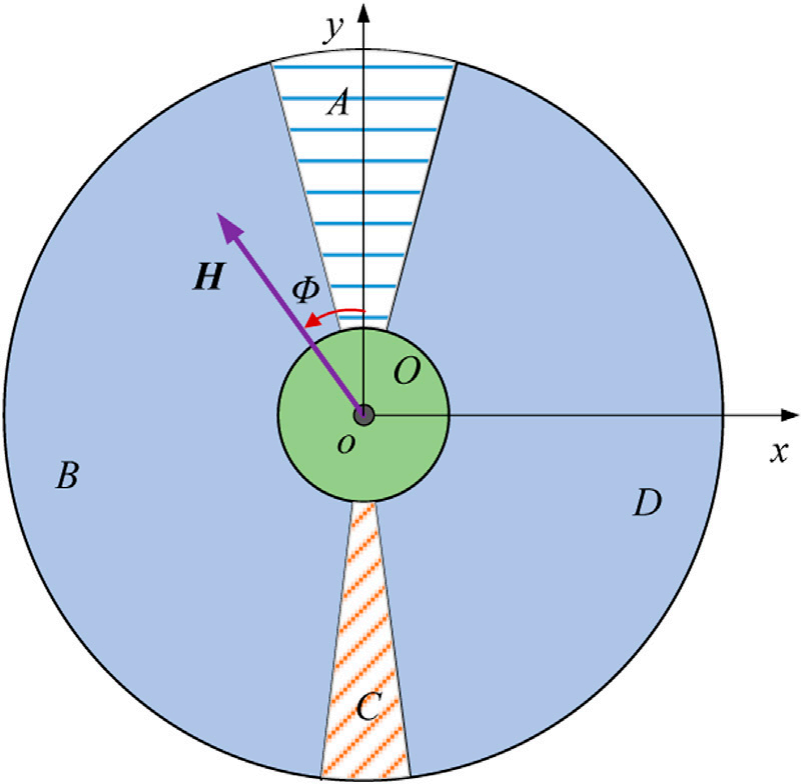
Definition of the five regions OABCD.

2) signal collection of the lever vector, as shown in Figure 6, obtain the direction and magnitude of the vector, save the data in time order;

3) predict the motion intention via the current region and change of the region, the relation between the change rule and the motion intention is shown in Table 1.

**TABLE 1:** Basic movement pattern of the robotic walker.

Region	O	A	B	C	D
O	—	Forward	Turn left	Backward	Turn right
A	Stop	—	Front-left	—	Front-right
B	Stop	Forward	—	Stop	—
C	Stop	—	Stop	—	Stop
D	Stop	Forward	—	Stop	—

The basic movement pattern of the robotic walker is shown in Table 1, forward, backward, turn left, turn right, front-left, front-right, and stop respectively. When the change rule is of the O-A, B-A, or D-A, the system predicts the human intends to move forward; when the change rule is of the O-C, the system predicts the human intends to move backward; when the change rule is of the O-B, the system predicts the human intends to turn left; when the change rule is of the O-D, the system predicts the human intends to turn right; when the change rule is of the A-B, the system predicts the human intends to move to front-left; when the change rule is of the A-D, the system predicts the human intend to move to front-right; and the walker will stop with other conditions. Besides, when the interaction forces show abnormal values or rapid change, or the real sense camera detects a dangerous expression, or the emergency stop button is pressed, the walker performs the Stop action.

The decision rule by the interaction forces has been discussed in the previous paper (Ji et al., 2020). For facial recognition, we use a real sense camera to detect the Yaw, Roll, and Pitch angle of the head, the angle feature Ε is used to assist the prediction. When the angle feature $Ε\in \left[\begin{array}{cc} -\pi /12 & \pi /12 \end{array}\right]$ , the system predicts the human intends to move forward, and we define that Ε=1 ; when the angle feature $Ε\in \left[\begin{array}{cc} -\pi /2 & -\pi /12 \end{array}\right]$ , the system predicts the human intends to turn left, and we define that Ε=2 ; when the angle feature $Ε\in \left[\begin{array}{cc} \pi /12 & \pi /2 \end{array}\right]$ , the system predicts the human intends to turn right, and we define that Ε=3 ; we define that Ε=0 with other conditions. In practice, apply the comprehensive methods to improve the recognition precision, and classify the move patterns via the multimodal fusion.

Then the controller calculates the output based on the classification results and interaction single, the function relationship can be expressed as Eq. 17:

$$f\left(v_{R},\omega_{R},r_{R}\right)=g\left(H,F,E\right)$$ (17)

After the signal has been processed, we define the dead zone to improve stability, then the robot velocity in the local coordinate system can be expressed as Eqs 18, 19:

$$v_{R}=\left\{\begin{array}{c} k_{p}Η+k_{d}\dot{Η}+k_{F}\sum_{i=1}^{2}F_{i},E>0\\ 0,E\leq 0 \end{array}\right.$$ (18)

$$\omega_{R}=\left\{\begin{array}{c} k_{p}Η\sin\Phi +k_{d}\dot{Η}\sin\Phi +k_{F}∆F_{i},E>0\\ 0,E\leq 0 \end{array}\right.$$ (19)

$$r_{R}=\frac{m}{\left|Η\sin\Phi \right|}-1,\in \left(0,+\infty \right)$$ (20)

where $k_{p}$ and $k_{d}$ are the PD gain coefficient, and $k_{F}$ is the gain coefficient of the interaction force.

Then by Eq. 12 to calculate the robot velocity in the global coordinate system $t_{M}$ , which is used to obtain the motion trail in the plane OXY, so we can compare the reference path and the actual path, and evaluate the effectiveness of the control method.

## 4 Experiment and verification

To prove the effectiveness of the proposed control method, a preliminary experiment with three healthy volunteers was carried out in this study. In the preliminary experiment, three healthy volunteers were asked to finish a task within the required time. Three kinds of trajectories were chosen to simulate the ADL task which were printed on the ground, and then the actual path data was recorded by the Programmable Logic Controller (PLC).

### 4.1 Experimental setup

The robotic walker was tested with the proposed control method and three health volunteers. The “∞” path, “○” path, and “□” path were used to test if the robotic walker could allow the volunteers to walk naturally. The experiment was carried out to evaluate the control performance by asking the three healthy volunteers to walk along the three given paths respectively. Three healthy adults were selected for the experiment study, and three men with an average age of 30.7, height of 173.3 cm, and weight of 71.7 kg were involved. Before the experiment, the volunteer wore a harness to connect with the robotic walker and adjusted the pelvic width. Inclusion criteria are no abnormalities in the nervous system, muscle-bone system, or found during physical examination, and having had no special balance training previously. A commonly used “∞” curve was firstly painted in black on the ground as the target path that the volunteers were asked to follow, and then the “∞” curve was replaced with a “○” and “□” curve, respectively. Each person had one chance to try the three paths and the experiment results were recorded by the encoders of the three omni wheels. In the experiment, the volunteers were asked to familiarize the operation of the robotic walker for 5 min and then finish the task within 60 s.

### 4.2 Data processing

For the tracking error analysis, the three target paths were mathematized in MATLAB to ensure the consistency between the painted curve and the mathematized curve. The actual position and orientation of the robotic walker were calculated by the three encoders of the driving wheel, which were calculated by the previously derived formulas. Then calculating the error between the actual path and reference pose samples, for comparison, the amount of the discrete point was processed into consistent. The normalized integral square error (ISE) cost function was used to evaluate the path-tracking error.

**FIGURE 7:**
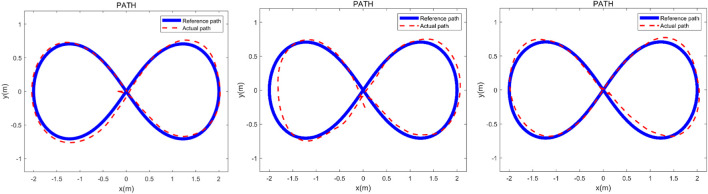
Verification experiment to finish the “∞” curve with three healthy adults. The heavy line is the reference path and the dashed line represents the actual path.

**FIGURE 8:**
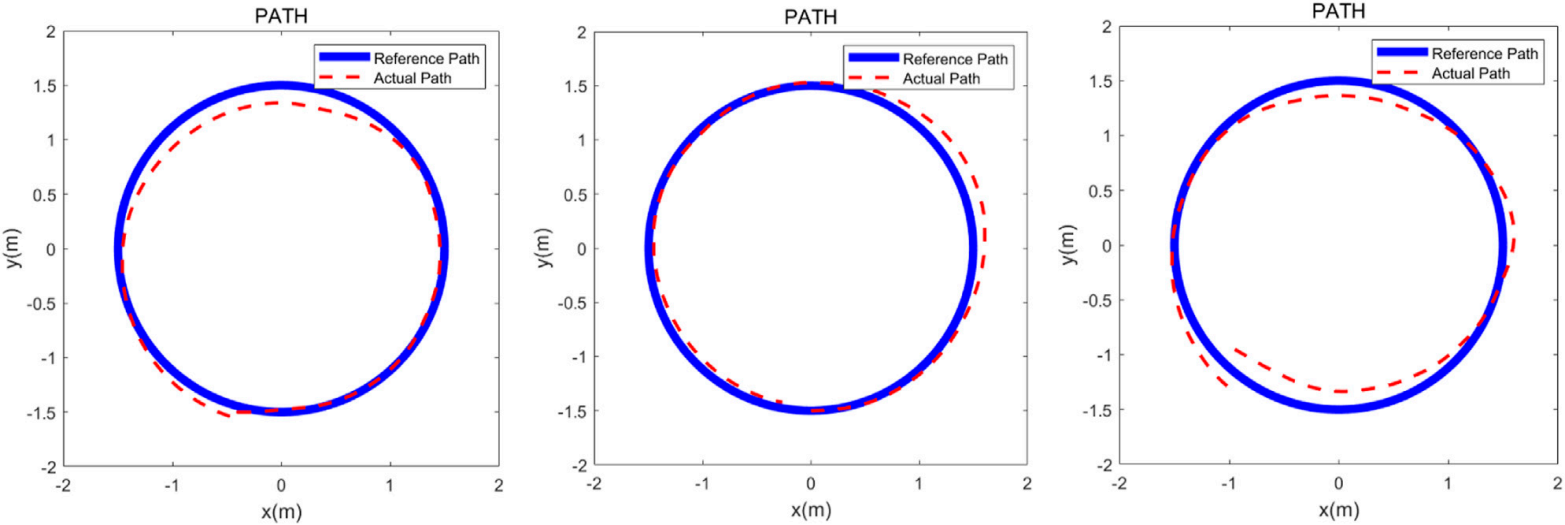
Verification experiment to finish the “○” curve with three healthy adults. The heavy line is the reference path and the dashed line represents the actual path.

**FIGURE 9:**
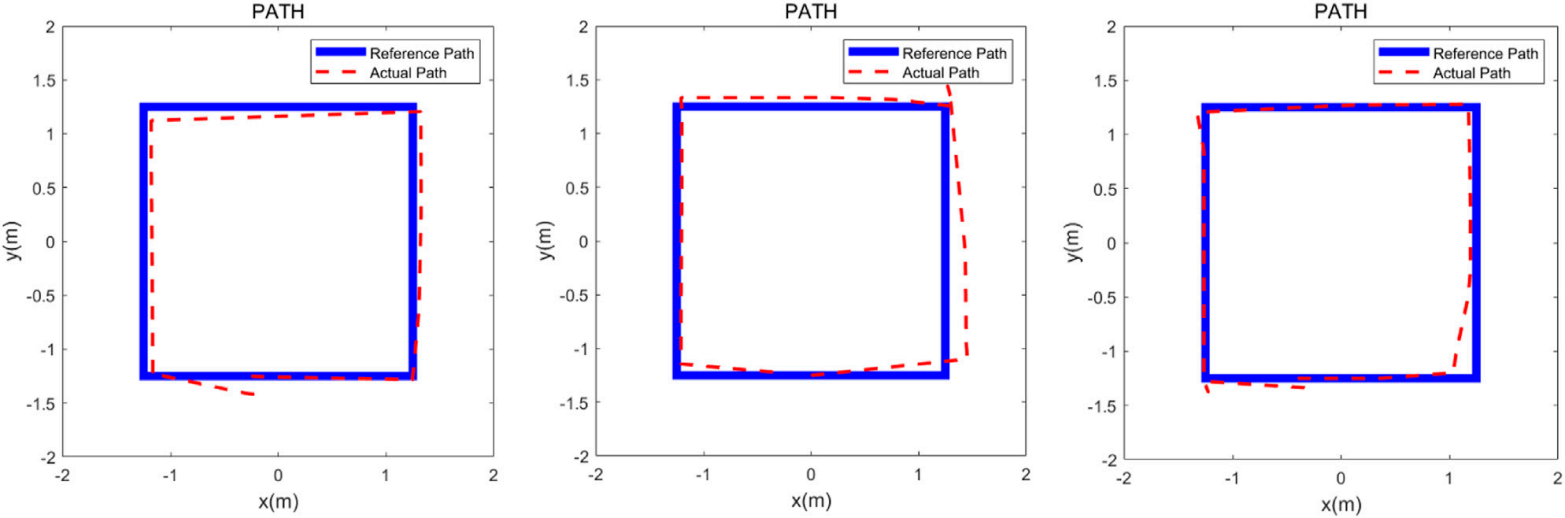
Verification experiment to finish the “□” curve with three healthy adults. The heavy line is the reference path and the dashed line represents the actual path.

Descriptive and analytical statistics were performed by the SPSS 22.0 and MATLAB 2016b. The actual paths were time normalized to 100% reference path. The error mean and standard deviation of position and orientation is defined by the difference between the target path and the reference path. The experimental results are shown in Figures 7–9.

## 5 Discussion

The three experimental results are shown in Figures 7–9, the volunteers finished the task within the prescribed time, it can be seen that the volunteers can follow the three given paths within an acceptable error range, and the “∞” curve is the most difficult task, indicating that the robotic walker allows the person to walk naturally under the *m*HRI and control with very minimal effort. As shown in Figure 7, the initial point and the endpoint are in the center of the curve, and volunteer 1 and volunteer 2 showed relatively big errors, and the ISE results are 2.69, 3.84, and 3.43 respectively, compared with the previous experimental results, the proposed method has a significant improvement. For the verification experiment to finish the “○” curve, the volunteers easily finished the task within the prescribed time, and the ISE results are 2.85, 2.15, and 3.76 respectively. For the “□” curve experiment, the volunteers easily finished the task within the prescribed time, and the ISE results are 2.44, 2.96, and 1.98 respectively. The results show that subjects can operate the walker to follow the prescribed curve, and it is evident that the walker can recognize the motion intent accurately and the volunteers can control the walker to fulfill the given task. This tracking experiment paves the way for the clinical application.

## 6 Conclusion

The present work demonstrates that the robotic walker is capable of intent recognition with the proposed *m*HRI system, in the ADL assistance, the robotic walker has the potential to reduce the stress on relatives of the patient. The proposed control algorithm for the motion control is derived via the robot kinematics and multimodal fusion human-robot interaction and proved to be effective in the pursuit movement by the preliminary experiment with three health volunteers. The experiment result shows that the robotic walker can effectively predict the user’s movement intention and provide appropriate output velocity to track. The RRW system may be used to improve the gait function of stroke survivors which is crucial to their quality of life.

## Data Availability

The original contributions presented in the study are included in the article/supplementary material, further inquiries can be directed to the corresponding authors.

## References

[B1] Calabrò R. S. Sorrentino G. Cassio A. Mazzoli D. Andrenelli E. Bizzarini E. (2021). Robotic-assisted gait rehabilitation following stroke: a systematic review of current guidelines and practical clinical recommendations. Eur. J. Phys. Rehabil. Med. 57 (3), 460–471. 10.23736/s1973-9087.21.06887-8 33947828

[B2] Capecci M. Pournajaf S. Galafate D. Sale P. Le Pera D. Goffredo M. (2019). Clinical effects of robot-assisted gait training and treadmill training for Parkinson's disease. A randomized controlled trial. Ann. Phys. Rehabil. Med. 62 (5), 303–312. 10.1016/j.rehab.2019.06.016 31377382

[B3] Chen Y. Chen Y. Zheng K. Dodakian L. See J. Zhou R. (2020). A qualitative study on user acceptance of a home-based stroke telerehabilitation system. Top. Stroke Rehabil. 27 (2), 81–92. 10.1080/10749357.2019.1683792 31682789 PMC7012699

[B4] Cifuentes C. A. Anselmo F. (2016). Human-robot interaction strategies for walker-assisted locomotion.

[B5] Feigin V. L. Brainin M. Norrving B. Martins S. Sacco R. L. Hacke W. (2022). World stroke organization (WSO): global stroke fact sheet 2022. Int. J. Stroke 17 (1), 18–29. 10.1177/17474930211065917 34986727

[B6] Frizera A. Ceres R. Frizera-Neto A. Ceres R. Rocon E. Pons J. L. (2011). Empowering and assisting natural human mobility: the simbiosis walker. Int. J. Adv. Robot. Syst. 80 (3), 29–50. 10.5772/10666

[B7] GBD Stark B. A. Johnson C. O. Roth G. A. Bisignano C. Abady G. G. (2021). Global, regional, and national burden of stroke and its risk factors, 1990–2019: a systematic analysis for the Global Burden of Disease Study 2019. Lancet Neurol. 20 (10), 795–820. 10.1016/s1474-4422(21)00252-0 34487721 PMC8443449

[B8] Haoyong Yu. Spenko M. Dubowsky S. (2003). An adaptive shared control system for an intelligent mobility aid for the elderly. Auton. Robots 15 (1), 53–66. 10.1023/a:1024488717009

[B9] Horii T. Nagai Y. (2021). Active inference through energy minimization in multimodal affective human-robot interaction. Front. Robot. AI 8, 684401. 10.3389/frobt.2021.684401 34901166 PMC8662315

[B10] Hurt C. P. Burgess J. K. Brown D. A. (2015). Limb contribution to increased self-selected walking speeds during body weight support in individuals poststroke. Gait Posture 41 (3), 857–859. 10.1016/j.gaitpost.2015.02.004 25770079 PMC4408234

[B11] Jarva E. Mikkonen K. Tuomikoski A. M. Kääriäinen M. Meriläinen M. Karsikas E. (2021). Healthcare professionals' competence in stroke care pathways: a mixed-methods systematic review. J. Clin. Nurs. 30 (9-10), 1206–1235. 10.1111/jocn.15612 33350004

[B12] Ji J. Chen W. Wang W. Xi J. (2021). Design and control of an omni-directional robotic walker based on human–machine interaction. IEEE Access 9, 111358–111367. 10.1109/ACCESS.2021.3103202

[B13] Ji J. Guo S. Xi F. Zhang L. (2020). Design and analysis of a smart rehabilitation walker with passive pelvic mechanism. J. Mech. Robotics 12 (3). 10.1115/1.4045509

[B14] Lesaine E. Francis-Oliviero F. Domecq S. Bijon M. Cetran L. Coste P. (2022). Effects of healthcare system transformations spurred by the COVID-19 pandemic on management of stroke and STEMI: a registry-based cohort study in France. BMJ Open 12 (9), e061025. 10.1136/bmjopen-2022-061025 PMC9494013 36130741

[B15] Mirelman A. Bonato P. Camicioli R. Ellis T. D. Giladi N. Hamilton J. L. (2019). Gait impairments in Parkinson's disease. Lancet Neurol. 18 (7), 697–708. 10.1016/s1474-4422(19)30044-4 30975519

[B16] Mojadidi M. K. Mahmoud A. N. Elgendy I. Y. (2017). Percutaneous patent foramen ovale closure for cryptogenic stroke: learning from clinical trial and error. J. Thorac. Dis. 9 (11), 4222–4225. 10.21037/jtd.2017.09.153 29268477 PMC5721015

[B17] Moreira R. Alves J. Matias A. Santos C. (2019). Smart and assistive walker - ASBGo: rehabilitation robotics: a smart-walker to assist ataxic patients. Adv. Exp. Med. Biol. 1170, 37–68. 10.1007/978-3-030-24230-5_2 32067202

[B18] Morioka K. Lee J. H. Hashimoto H. (2004). Human-following mobile robot in a distributed intelligent sensor network. IEEE Trans. Ind. Electron. 51 (1), 229–237. 10.1109/tie.2003.821894

[B19] Nam K. Y. Kim H. J. Kwon B. S. Park J. W. Lee H. J. Yoo A. (2017). Robot-assisted gait training (Lokomat) improves walking function and activity in people with spinal cord injury: a systematic review. J. Neuroeng Rehabil. 14 (1), 24. 10.1186/s12984-017-0232-3 28330471 PMC5363005

[B20] Ohnuma T. Lee G. Chong N. Y. (2017). Development of JARoW-II active robotic walker reflecting pelvic movements while walking. Intell. Serv. Robot. 10, 95–107. 10.1007/s11370-016-0212-7

[B21] Pons J. Ceres R. Calderón L. (2008). “Chapter introduction to wearable robots and exoskeletons,” in Wearable robots: biomechatronic exoskeletons (Wiley), 1–5.

[B22] Reisman D. S. Bastian A. J. Morton S. M. (2010). Neurophysiologic and rehabilitation insights from the split-belt and other locomotor adaptation paradigms. Phys. Ther. 90 (2), 187–195. 10.2522/ptj.20090073 20023001 PMC2816031

[B23] Seo K. H. Lee J. J. (2009). The development of two mobile gait rehabilitation systems. IEEE Trans. Neural Syst. Rehabil. Eng. 17 (2), 156–166. 10.1109/tnsre.2009.2015179 19228564

[B24] Sharma R. Pavlovic V. I. Huang T. S. (1998). Toward multimodal human-computer interface, in Proc. IEEE, 860(5): 853–869. 10.1109/5.664275

[B25] Sierra M. Sergio D. Garzón M. Cifuentes C. A. (2019). Human–robot–environment interaction interface for smart walker assisted gait: AGoRA walker. Sensors 19 (13), 2897. 10.3390/s19132897 31262036 PMC6650898

[B26] Sierra M. S. D. Jimenez M. F. Múnera M. C. Bastos T. Frizera-Neto A. Cifuentes C. A. (2019). A therapist helping hand for walker-assisted gait rehabilitation: a pre-clinical assessment," 2019 ieee 4th Colombian conference on automatic control. Medellin, Colombia: CCAC, 1–6.

[B27] Su H. Qi W. Chen J. Yang C. Sandoval J. Laribi M. A. (2023). Recent advancements in multimodal human-robot interaction. Front. Neurorobot 17, 1084000. 10.3389/fnbot.2023.1084000 37250671 PMC10210148

[B28] Valadão C. Caldeira E. Bastos-Filho T. Frizera-Neto A. Carelli R. (2016). A new controller for a smart walker based on human-robot formation. Sensors 16, 1116. 10.3390/s16071116 27447634 PMC4970159

[B29] Wang W. Gong T. Song Z. Wang Z. Ji J. (2023). Simulation study on assist-as-needed control of a rehabilitation robotic walker. Technol. Health Care 31 (S1), 293–302. 10.3233/thc-236025 PMC10200137 37066930

[B30] Zhao X. Zhu Z. Liu M. Zhao C. Zhao Y. Pan J. (2020). A smart robotic walker with intelligent close-proximity interaction capabilities for elderly mobility safety. Front. Neurorobot 14, 575889. 10.3389/fnbot.2020.575889 33192437 PMC7642877

